# Enisamium Inhibits SARS-CoV-2 RNA Synthesis

**DOI:** 10.3390/biomedicines9091254

**Published:** 2021-09-17

**Authors:** Stefano Elli, Denisa Bojkova, Marco Bechtel, Thomas Vial, David Boltz, Miguel Muzzio, Xinjian Peng, Federico Sala, Cesare Cosentino, Andrew Goy, Marco Guerrini, Lutz Müller, Jindrich Cinatl, Victor Margitich, Aartjan J. W. te Velthuis

**Affiliations:** 1 Istituto di Ricerche Chimiche e Biochimiche “G. Ronzoni”, Via Giuseppe Colombo 81, 20133 Milano, Italy; elli@ronzoni.it (S.E.); sala@ronzoni.it (F.S.); cosentino@ronzoni.it (C.C.); guerrini@ronzoni.it (M.G.); 2 Institute of Medical Virology, University Hospital Frankfurt am Main, Goethe University, Paul-Ehrlich-Straße 40, 60596 Frankfurt am Main, Germany; denisa.bojkova@kgu.de (D.B.); marco.bechtel94@gmx.de (M.B.); 3 Division of Virology, Department of Pathology, Addenbrooke’s Hospital, University of Cambridge, Hills Road, Cambridge CB2 2QQ, UK; tv293@cam.ac.uk; 4 IIT Research Institute, 10 W 35th St, Chicago, IL 60616, USA; dboltz@iitri.org (D.B.); mmuzzio@iitri.org (M.M.); xpeng@iitri.org (X.P.); 5 Farmak Joint Stock Company, Kyrylivska Street, 04080 Kyiv, Ukraine; a.goy@farmak.ua (A.G.); v.margitich@farmak.ua (V.M.); 6 Regenold GmbH, Zöllinplatz 4, 79410 Badenweiler, Germany

**Keywords:** COVID-19, SARS-CoV-2, molecular dynamics simulation, RNA polymerase, FAV00A, Amizon

## Abstract

Pandemic SARS-CoV-2 causes a mild to severe respiratory disease called coronavirus disease 2019 (COVID-19). While control of the SARS-CoV-2 spread partly depends on vaccine-induced or naturally acquired protective herd immunity, antiviral strategies are still needed to manage COVID-19. Enisamium is an inhibitor of influenza A and B viruses in cell culture and clinically approved in countries of the Commonwealth of Independent States. In vitro, enisamium acts through metabolite VR17-04 and inhibits the activity of the influenza A virus RNA polymerase. Here we show that enisamium can inhibit coronavirus infections in NHBE and Caco-2 cells, and the activity of the SARS-CoV-2 RNA polymerase in vitro. Docking and molecular dynamics simulations provide insight into the mechanism of action and indicate that enisamium metabolite VR17-04 prevents GTP and UTP incorporation. Overall, these results suggest that enisamium is an inhibitor of SARS-CoV-2 RNA synthesis in vitro.

## 1. Introduction

Severe acute respiratory coronavirus 2 (SARS-CoV-2) is an important human pathogen and the causative agent of coronavirus disease 2019 (COVID-19). Vaccines are available to prevent the spread of SARS-CoV-2, and several antiviral strategies, such as treatment with remdesivir or reconvalescent plasma, have received FDA approval or emergency use approval. However, the development of additional strategies remains necessary, because of the continuous emergence of variants of concern (VOC) and current antiviral treatments can only be delivered intravenously. A key target for novel drug screening is the RNA polymerase of SARS-CoV-2 [1,2,3,4].

SARS-CoV-2 is a betacoronavirus and contains a positive-sense, non-segmented RNA genome of around 30 kilobases [5,6]. The 5′ two-thirds of the viral genome encodes two overlapping open reading frames (ORFs), 1a and 1b, which are translated into two large polyproteins by host cell ribosomes. The two polyproteins are cleaved by intrinsic proteolytic activity to produce 16 non-structural proteins (nsps). Nsp12 is the RNA-dependent RNA polymerase that copies and transcribes the SARS-CoV-2 genome [7,8]. Nsp12 requires nsp7 and nsp8 for processivity in vitro [9]. The structures of nsp12/7/8 from SARS-CoV and SARS-CoV-2 were solved by cryo-EM [10,11,12].

**Figure 1:**
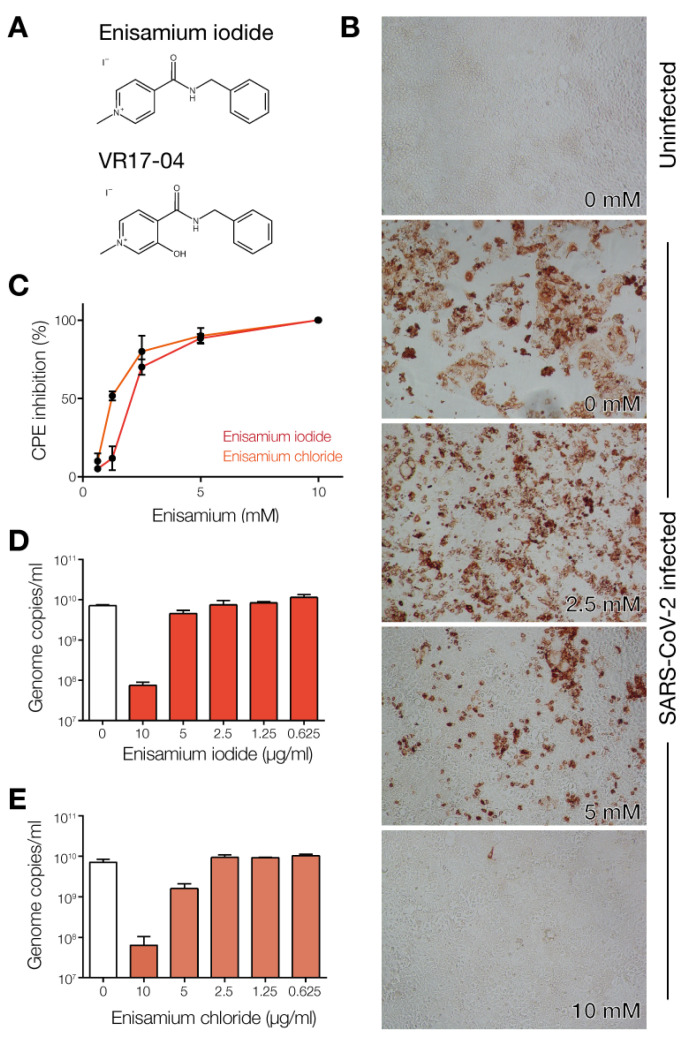
Enisamium inhibits SARS-CoV-2 infection of Caco-2 cells in vitro. (**A**) Chemical structures of enisamium iodide (FAV00A) and VR17-04. The chemical structure of FAV00B is identical to FAV00A except that chloride ions are present instead of iodide. (**B**) Inhibition of SARS-CoV-2 N expression by enisamium chloride in Caco-2 cells. (**C**) Inhibition of SARS-CoV-2 cytopathic effect by enisamium iodide and chloride in Caco-2 cells. (**D**) Effect of enisamium iodide or (**E**) enisamium chloride on SARS-CoV-2 infected Caco-2 cells.

Various nucleoside analogues and small molecule inhibitors were identified as potential inhibitors of the SARS-CoV-2 nsp12/7/8 complex [1,2,3]. One of the drugs highlighted by the World Health Organization as a candidate therapeutic against SARS-CoV-2 is enisamium (4-(benzylcarbamoyl)-1-methylpyridinium); Figure 1A). Enisamium is licensed for use against influenza in 11 countries and it was shown to reduce virus shedding and improve influenza patient recovery [13]. A recent study found that enisamium is hydroxylated in humans and human lung cells to a compound called VR17-04 (Figure 1A) [13]. VR17-04 inhibits the activity of the influenza virus RNA polymerase [13].

In this study, we show that enisamium inhibits coronavirus growth on Caco-2 and normal human bronchial epithelial (NHBE) cells. We also demonstrate that enisamium inhibits the minimal viral RNA polymerase complex in a mini-genome assay. Molecular dynamics (MD) simulation analysis suggests that VR17-04 reversibly binds to the exposed cytosine or adenine bases, preventing incorporation of GTP and UTP into the nascent strand. Overall, these results suggest that enisamium prevents SARS-CoV-2 RNA synthesis by inhibiting nascent strand elongation.

## 2. Materials and Methods

### 2.1. SARS-CoV-2 Infections

Confluent layers of Caco-2 cells in 96-well plates were treated with serial dilutions of enisamium iodide (laboratory code FAV00A, trade name Amizon^®^) or enisamium chloride (laboratory code FAV00B) 6 h prior to infection. The cells were infected with SARS-CoV-2 at multiplicity of infection 0.01 for 1 h, and compound reapplied following virus removal. At 48 h post infection, the cytopathic effect was recorded by examination of the infected cultures by light microscopy and supernatant collected to quantify virus RNA by RT-qPCR as described previously, using nsp12-specific primers 5′-GTGARATGGTCATGTGTGGCGG-3′ and 5′-CARATGTTAAASACACTATTAGCATA-3′ [13,14,15,16,17,18,19,20,21]. Cells were fixed with acetone/methanol (40:60) and immunostained using a SARS-CoV-2 nucleoprotein monoclonal antibody (1:500, Sinobiological, Cat #40143-R019-100 µL). Staining was detected using a peroxidase conjugated anti-rabbit secondary antibody (1:1000, Dianova) and the addition of AEC substrate.

### 2.2. HCoV-NL63 NHBE Infections

MatTek’s EpiAirway System (MatTek; Ashland, MA, USA) consisted of differentiated NHBE cells that were cultured to form a multilayered, highly differentiated model that closely resembles the epithelial tissue of the respiratory tract. The cells from a single donor (No. 9831) were used for assay consistency. The apical surface of the cells was exposed to a humidified 95% air, 5% CO_2_ environment. The basolateral medium was changed, and the mucin layer was washed every 24–48 h. NHBE cells were inoculated via the exposure of the apical side to HCoV-NL63. After 1 h of incubation with virus in a water-jacketed 37 °C incubator with a 5% CO_2_ supply, the viral inoculum was removed from the cells. The apical side of the cells was washed once prior to infection. After viral inoculation, enisamium chloride or control media were added to the apical side of the cells and the basal media compartment and incubated with the cells for 1 h. After a 1-hour incubation, the drug containing media was removed from apical and basal chambers. Growth medium alone or growth medium with enisamium chloride were added to the bottom chamber, and cells were incubated for 48 h. At the termination of the experiments, cells were washed twice, then 1 mL Trizol (Thermo Fisher, Waltham, MA, USA) was added to each well for RNA isolation. Total RNA was isolated from cells using Trizol per the manufacturer’s instruction. Two-step RT-qPCR was performed using HCoV-NL63 N gene-specific primers/probe (forward primer: 5′-TGGTGTTGTTTGGGTTGCTA-3′, reverse primer 5′-GCTCTGGAGGCAAAGCAATA-3′, double-quenched probe: 5′-FAM/CGCAAACGT/ZEN/AATCAGAAACCTTTGGA/IABKFQ-3′), GAPDH was analyzed at the same time with GAPDH-specific primers (5′-GTTCGACAGTCAGCCGCATC-3′ and 5′-AGTTAAAAGCAGCCCTGGTGA-3′) by RT-qPCR and served as a reference gene for normalization.

### 2.3. SARS-CoV-2 Minigenome Assay

The SARS-CoV-2 mini-genome assay was performed as described previously [14]. Briefly, approximately 2 × 10^5^ HEK 293T cells were transfected with 200 ng pcDNA6.B-nsp7-flag, pcDNA6.B-nsp8-flag, and pcDNA6.B-nsp12-flag, and 25 ng linear pPolI-SARS-CoV2-NLuc-N plasmid using Lipofectamine 2000 (Thermo Fisher). Cells were collected 24 h post-transfection and analyzed using a Nano-Glo dual-luciferase reporter assay (Catalogue number N1610; Promega; Madison, WI, USA) according to the manufacturer’s protocol and a Glomax navigator microplate Luminometer (Promega). For each condition, three biological replicates were conducted.

### 2.4. NOE NMR Spectra Measurement

The VR17-04 (2 mg) was dissolved in 0.6 mL D2O/water solution (5% D2O) or DMSO. NMR experiments were performed at 500 MHz at 277 K. For NOESY experiments (noesygpph) 128 transients were collected for each free-induction decay, using a mixing time of 300 ms and 20 sec of relaxation delay. The NOESY experiment (matrix 1024 × 320 points) was zero-filled to 2K × 2K before Fourier transformation. Measurements were taken on a Bruker 500 HD NMR spectrometer equipped with a 5 mm BB probe.

### 2.5. Conformational Characterization and Geometry Optimization

The VR17-04 and enisamium conformational characterization and geometry optimization was performed using the quantum chemical approach DFT B3LYP/6-31G*. The quantum chemistry software GAUSSIAN 2016 was used at this stage [22]. The explored conformations of VR17-04 and enisamium are characterized by the dihedral angles defined by the following consecutive four atoms indicated in bold: φ_0_ (**C**(Ar)H-**C**(Ar)-**C**(CH_2_)-**N**) φ_1_ (**C**(Ar)-**C**(CH_2_)-**N**-**H**), φ_2_ (**H**-**N**-**C**-**O**), φ_3_ (**O**-**C**-**C**(Ar)-**C**(Ar)OH/H). The lowest energy conformation of VR17-04 (φ_3_ *eclipsed*) and enisamium were selected for further docking and for partial charge estimation (Table 1).

### 2.6. Molecular Docking

The docking simulation was performed using Autodock 4.2 software [23]. The geometry of the ligands was previously optimized by DFT B3LYP/6-31G*. The geometry of the receptor RdRp was extracted from the PDB 7bv2 [24]. The catalytic site of the nsp12/7/8 complex include the template RNA strand, the nascent strand RNA, the pyrophosphate moiety [O_3_P-O-PO_3_]^−4^ (pyr) and two Mg^+2^ ions, whose positions were included in 7BV2. The two Zn^+2^ ions that were co-crystallized in 7BV2 were also included in the models. The co-crystallized inhibitor remdesivir monophosphate was removed from the complex, while its position and the contacts with the template RNA through the unpaired uracil base in the +1 position were used to guide the molecular docking. The unpaired uracil base was subsequently mutated in cytosine (Cyt) or adenine (Ade) in Pymol 2.3.4 (Schrodinger Inc; New York, NY, USA), generating two different complexes, identified as nsp12/7/8(Cyt) and nsp12/7/8(Ade), respectively. Next, Gasteiger charges were calculated for both ligands (VR17-04, enisamium) and the receptor complexes, and used as parameters of the docking simulation [25]. In the docking simulation VR17-04 and enisamium were described by five and four rotational degrees of freedom, respectively. The docking gridbox was built by orthogonal hedges of length between 60 and 80 points. The gridbox centre was set to the NH2- group of the target cytosine or adenine residues of the template RNA strand, respectively, and further set-up to fit the space between the R555 and K545, and the uracil of the nascent RNA strand. The docking runs used the default genetic algorithm search, with parameters: number of GA runs, population size, max number of energy evaluation, and max number of generations set as 100, 2000, 2.5 × 10^7^, 270,000. At each run the docking solutions were clustered using a tolerance RMSD = 2.0 Å. Three different docking simulations were run: VR17-04 and enisamium were docked on to nsp12/7/8(Cyt), while, for comparison, VR17-04 was further docked on nsp12/7/8(Ade). The docking solutions were selected based on two criteria. The first criterium was the possibility to form a Watson–Crick base-pair interaction between VR17-04 (or enisamium) and the unpaired cytosine or adenine. The second criterium was based on the possibility to figure out interferences of the ligand with the catalytic mechanism, for example by interaction with key residues of the nsp12/7/8 complex. The selected poses were further ranked by a preliminary MD simulation (approximately 50 ns) in explicit solvent, to predict the stability of the interaction, and to estimate the Poisson–Boltzmann free energy of binding, this last property was used to obtain a final selection and ranking of the poses.

### 2.7. Molecular Dynamic Simulation

Explicit solvent MD simulations were run using NAMD 2.12 [26] software and the Amber force-field (ff14SB) [27]. The TIP3P [28] water solvent model was used. The t-leap application of the Ambertools 14.0 package [27] was applied to generate the topology, the parameters, and the coordinate files of the macromolecular complex simulated. The coordinates of the macromolecular elements of the nsp12/7/8 complex: nsp12, nsp8, nsp7, pyr, template RNA, and nascent strand RNA, were extracted from PDB 7bv2. The geometries of VR17-04, enisamium (before docking), and pyr were optimized using the quantum chemical approach DFT B3LYP/6-31G*; the corresponding partial charges were estimated by fitting the electrostatic potential that was calculated to the B3LYP/6-31G*/RHF/6-31G* model. This procedure is in accordance with the standard required by the Amber force-field. The quantum chemistry software GAUSSIAN 2016 was used at this stage [22]. The Amber atomtypes (parm10.dat) were selected for VR17-04, enisamium, and pyr (antechamber application, Ambertools 14.0). Three macromolecular complexes indicated as VR1704-nsp12/7/8(Cyt), enisamium-nsp12/7/8(Cyt), and VR1704-nsp12/7/8(Ade), were solvated by a 15 Å wide layer of TIP3P water molecules in each X, Y, Z direction; the orthogonal simulation box was built with hedges of approximate length 116, 116, 127 Å. The non-bond electrostatic and dispersive interactions were described by the standard cut-off technique (12.0 Å). Before running MD simulations, each simulation cell box was energy minimized by running 200 K steps of the default energy minimization algorithm, as implemented in NAMD. The MD simulations were run by fixing the number of particles (N), the absolute temperature (T), and the pressure (P) applied to the cell hedges. The absolute temperature was 300 K and maintained with a Lowe–Andersen thermostat, while the pressure on the cell box hedges was set as P = 1.01325 bar and preserved by the Nosé–Hoover–Langevin piston algorithm. The first MD simulation stage was run to adjust the simulation cell box density, allowing the relaxation of all the inter-molecule distances, i.e., the solute–solute, solute–solvent, and solvent–solvent distances. The cell density equilibration stage was run to restrain the atoms of the solute to their initial position (energy minimized geometry of the complex) by applying a harmonic restraint. In this stage of cell density equilibration, the water molecules were left free to move. The harmonic restraint constant value was set initially at 1 Kcal mol^−1^ for each atom of the solute, and progressively reduced at 0.5 and 0.2 Kcal mol^−1^. The cell density equilibration stage was monitored by plotting the cell volume (Å^3^) vs. time (ns), until the cell volume fluctuations level off to a horizontal axis that corresponds to the average final volume of the cell. This stage required between 20 and 50 ns approximately and was further checked by the formation of a thin water shell that surrounded the face of the inhibitor molecule that was exposed to the empty catalytic cavity of the nsp12/7/8 complex. In the second stage of the MD simulation, the harmonic restraint was removed, and the inhibitor-nsp12/7/8 complex was allowed to equilibrate in the geometry and the relative position of their elements: inhibitor, template RNA, nascent strand RNA, pyr, Mg^+2^, and Zn^+2^. To monitor the equilibration of the position and orientation that VR17-04 (or enisamium) occupies in the +1 position of the catalytic cavity, the distances between the carbonyl oxygen of VR17-04 and the hydrogen (NH2-) of cytosine (or adenine), and between the hydroxyl group of VR17-04 and the nearby lone pair of cytosine nitrogen (or adenine), were plotted vs. simulation time. These distances provide a direct indication of the stability of the Watson–Crick base pair interaction that hold the inhibitor near the target base. The orientation of VR17-04 in the catalytic site of the nsp12 was also monitored by the coplanarity angle χ as defined by the following atoms in bold: C**O**-**N**H_2_-**N**-**O**H, in which C**O** and **O**H belong to VR17-04, while the remaining **N**H_2_ and **N** belong to the opposite cytosine (or adenine). Alternatively, the enisamium-cytosine pair requires the following atoms C**O**-**N**H_2_-**N**-**C**H to define χ; in this case C**O** and **C**H belong to enisamium, while **N**H_2_ and **N** belong to the opposite cytosine. In fact, values of this angle around ‘0’ indicate that the ‘base pair’ contact between VR1704 the cytosine (or adenine) is coplanar, a geometric condition favoring the Watson–Crick ‘base-pair’ interaction between VR17-04 and the base. VMD 1.9.3 [29] was used for the MD simulation trajectory visualization and image creation.

### 2.8. Estimation of Poisson-Boltzmann Free Energy of Binding

In a system that evolves in accord to the complex formation reaction [30].

(1) L+R ⇆L−R

The free energy change is calculated knowing only the initial and final state of the system:

(2) $$\Delta G^{Bind}=G\left(L-R\right)-G\left(L\right)-G\left(R\right)$$

The free energy decomposed in the following terms:

(3) $$G=\langle E_{MM}\rangle +\langle G_{sol}\rangle +\langle G_{noPol}\rangle -T\langle S_{MM}\rangle$$

The free energy change is then conveniently split:

(4) $$\Delta G^{Bind}=\Delta G_{PB}^{Bind}+\Delta S_{MM}$$

In Equation (3) the *E_MM_* corresponds to the potential energy of the system, as described by the force-field; *G_sol_* is the polar solvation energy, estimated by the Poisson–Boltzmann equation [31]; *G_nopol_* is the non-polar solvation energy, estimated by the solvent-accessible surface area, a method included in the MMPBSA. *T* is the absolute equilibrium temperature, while *S_MM_* is the molecular entropy of the system. The sum of the first three terms on the right-hand side of the Equation (3) is conveniently defined as the Poisson–Boltzmann free energy of binding Δ*G_PB_^Bind^* (see Equation (4)). To estimate the absolute value of the free energy of binding Δ*G^Bind^* the molecular entropy change *ΔS_MM_* is required. Since in this study two similar molecules, VR17-04 and enisamium, were compared for their ability to bind cytosine (+1 position of the catalytic site of nsp12), or alternatively two bound states VR17-04-cytosine or VR17-04-adenine were inquired, the respective entropy changes Δ*S_MM_* are considered similar. In this condition the Poisson–Boltzmann free energy of binding Δ*G_PB_^Bind^* could be used to rank the selected molecular recognitions and/or the poses of the docking experiment.

## 3. Results

### 3.1. Enisamium Inhibits SARS-CoV-2 Infection in Cell Culture

Previous experiments showed that enisamium (Figure 1A) can efficiently inhibit influenza virus replication in normal human bronchial epithelial (NHBE) cultures and A549 cells, and to a lesser extent in Caco-2 cells [13]. Previous experiments have also demonstrated that enisamium is not cytotoxic to these cells [13]. To test if enisamium can inhibit pandemic SARS-CoV-2 replication in cell culture, we first incubated Caco-2 cells, a standard cell line for SARS-CoV-2 infection in vitro, with enisamium iodide or enisamium chloride for 6 h and subsequently infected the treated cells with SARS-CoV-2. After 48 h, inhibition of viral infection was assessed by antigen staining for viral nucleoprotein expression and RT-qPCR for viral genome replication. We observed a significant reduction in both viral nucleoprotein expression (Figure 1B), a reduction in the cytopathic effect of the SARS-CoV-2 infection on Caco-2 cells (Figure 1C), and a 2-log reduction in the number of viral genome copies in the infected cells as a function of the enisamium concentration (Figure 1D,E). The IC_50_ for enisamium chloride in Caco-2 cells was 1.2 mM (~300 µg/mL), which is comparable to the inhibitory effect of enisamium on influenza A virus replication in Caco-2 cells [13].

**Figure 2:**
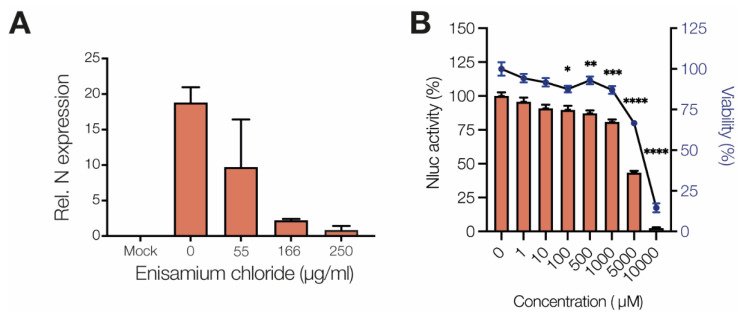
Enisamium inhibits HCoV-NL63 infection of NHBE cells and SARS-CoV-2 nsp12/7/8 activity in vitro. (**A**) Quantification of HCoV-NL63 N mRNA levels in NHBE cells infected with HCoV-NL63 after treatment with enisamium chloride. (**B**). Inhibition of SARS-CoV-2 nsp12/7/8 RNA polymerase complex activity by ensamium as measured with a mini-genome assay. Quantification is from *n* = 3 independently prepared reactions using the same nsp12/7/8 protein preparation. Error bars represent standard deviation. * *p* < 0.05, ** *p* < 0.01, *** *p* < 0.001, **** *p* < 0.0001.

To test if enisamium can inhibit coronavirus replication in NHBE cells, NHBE cells were incubated with enisamium iodide for 6 h and subsequently infected with alpha-coronavirus HCoV NL63. Analysis of the N gene RNA levels in infected NHBE cells showed a strong effect on viral RNA synthesis (Figure 2A) with an IC_50_ of ~60 µg/mL, implying that enisamium can inhibit coronavirus replication in NHBE cells in vitro.

### 3.2. Enisamium Inhibits SARS-CoV-2 nsp12/7/8 Activity

Previous experiments showed that enisamium can inhibit the influenza A virus RNA polymerase in vitro [13]. To test if enisamium can inhibit SARS-CoV-2 RNA synthesis in vitro, we used a recently established mini-genome assay [14]. This assay depends on the replication and transcription of an N-subgenomic mRNA that encodes the nanoluciferase gene by the minimal viral RNA polymerase complex nsp12/7/8 [14]. The addition of enisamium to the medium significantly inhibited the mini-genome signal in a concentration-dependent manner (Figure 2B).

### 3.3. Enisamium Adopts a Conformation in Solution That Would Be Compatible with Hydrogen Bond Formation

**Figure 3:**
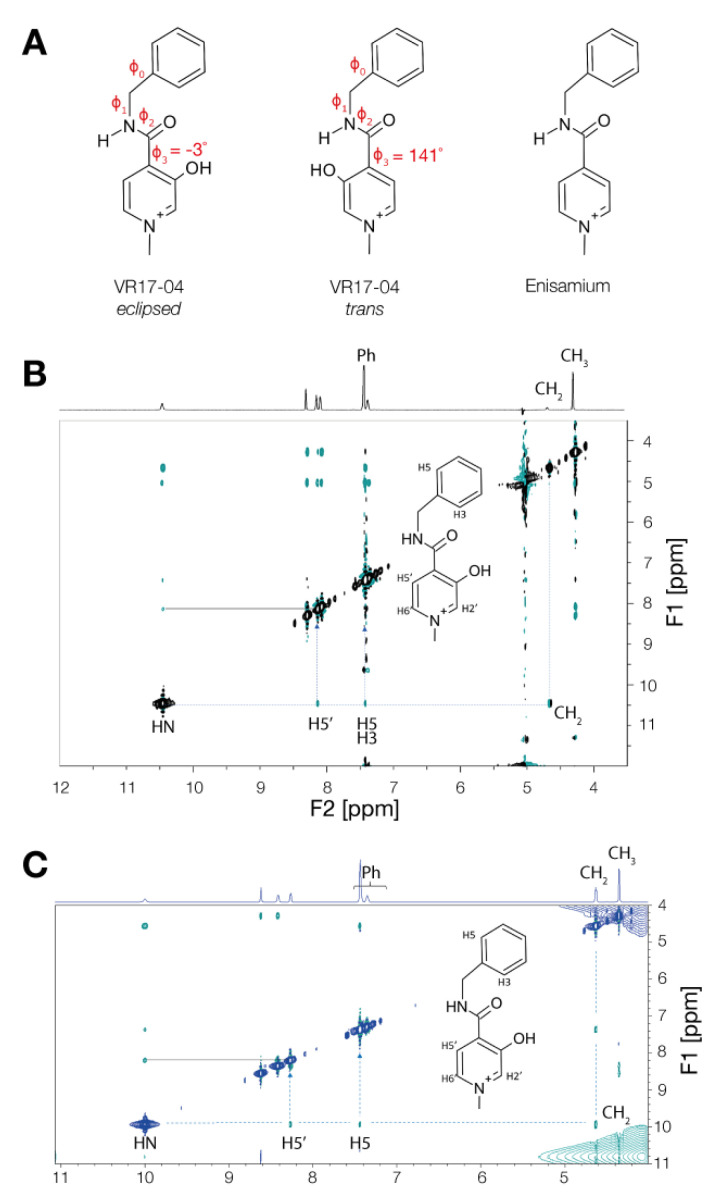
NMR spectrum of enisamium metabolite VR17-04. (**A**) Schematic of the *trans* and *eclipsed* conformations of VR17-04. Dihedral angles are indicated with φ. (**B**) 2D-NOESY and ^1^H proton spectra of VR17-04 acquired at 277 K in water. The NOE correlation between the HN and H5′ proton is highlighted with a dashed line. (**C**) 2D-NOESY and ^1^H proton spectra of VR17-04 acquired at 277 K in DMSO. The NOE correlation between the HN and H5′ proton is highlighted as in panel B.

We previously showed that enisamium is metabolized to VR17-04, and that VR17-04 can block influenza A virus RNA polymerase activity. A recent report also showed that VR17-04 can inhibit the activity of purified nsp12/7/8 in vitro [15]. The difference between enisamium and VR17-04 is the addition of a hydroxyl group to the methyl-pyridinium ring in VR17-04 (Figure 1A). The hydroxyl group allows VR17-04 to adopt two conformations that cannot be distinguished in enisamium. In particular, this hydroxyl group could be positioned parallel or opposite the carbonyl group that enisamium and VR17-04 share (Figure 3A), which we define as the *eclipsed* or *trans* conformations, respectively. In the *eclipsed* conformation, the hydroxyl and carbonyl groups of VR17-04 can form two hydrogen bonds using both a proton donor (H-O) and proton acceptor (O=C), potentially with a single interaction partner, while in the *trans* conformation, single hydrogen bonds could be made on both sides of VR17-04, potentially with two interaction partners. Quantum chemical calculations suggest that the *eclipsed* conformation of VR17-04 has a lower energy in unbound state than the *trans* conformation (Table 1), and thus that the *eclipsed* conformation would likely be favored in solution.

To investigate the mechanism by which enisamium metabolite VR17-04 inhibits the SARS-CoV-2 nsp12/7/8 complex, we needed more information on the conformation of VR17-04 in solution and in a bound state of VR17-04 with the RNA polymerase complex. The conformations of VR17-04 and enisamium are characterized by four dihedral angles: φ_0_, φ_1_, φ_2_ and φ_3_ (Figure 3A). Dihedral φ_3_ allows us to differentiate between the *trans* and *eclipsed* conformations of VR17-04, since its angle is 141° in the *trans* conformation and −3° in the *eclipsed* conformation (Figure 3A). Moreover, dihedral φ_3_ defines that HN and H5′ are proximal in *eclipsed* conformation, and opposite in the *trans* conformation (Figure 3B,C inset). To experimentally determine which conformation VR17-04 adopts in solution (unbound state), we measured the 1H NOESY spectrum of VR17-04 in water, in which SARS-CoV-2 nsp12/7/8 is active, and DMSO, in which VR17-04 is initially dissolved (Figure 3B,C). In both solutions, we observed a NOEs correlation peak between the protons HN and H5′, compatible with φ_3_ → 0° (Figure 3B,C). In addition, we observed a NOEs correlation between HN and CH_2_, and between HN and the ortho aromatic protons of the Ph group (H5, H3 in Figure 3C). The chemical shifts of the selected protons are reported in Table 2. Together these observations suggest that VR17-04 preferentially adopts an *eclipsed* conformation in an aqueous solution.

### 3.4. VR17-04 Forms Hydrogen Bonds with Cytosine and Adenosine in MD Simulations

**Figure 4:**
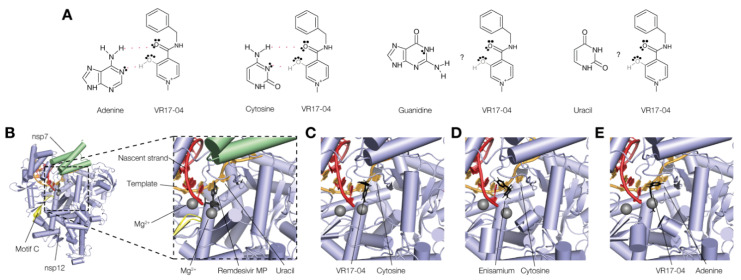
Molecular docking of VR17-04 binding into the nsp12/7/8 complex. (**A**) Schematic of putative hydrogen bond formation between cytosine and adenine bases with VR17-04. (**B**) Structure of the SARS-CoV-2 nsp12/7/8 complex bound to RNA and remdesivir monophosphate. Rendering based on PDB 7bv2. (**C**) Docking of VR17-04 binding to cytosine in nsp12 active site. (**D**) Docking of enisamium binding to cytosine in nsp12 active site. (**E**) Docking of VR17-04 binding to adenine in nsp12 active site.

Enisamium metabolite VR17-04 inhibits the activity of the influenza A virus [13] and SARS-CoV-2 nsp12 RNA polymerases (Figure 2B), suggesting that it targets a conserved site. As noted above, in the *eclipsed* conformation, the OH group on the methyl-pyridinium ring and the central CO group could form two hydrogen bonds with an interaction partner. This type of interaction is reminiscent of base-pairing between RNA or DNA strands, and we hypothesized that VR17-04 could form two hydrogen bonds with adenine and cytosine (Figure 4A). In this model, enisamium would only form one hydrogen bond with a cytosine or adenine in the template strand, resulting in a weaker interference with the RNA replication process. Interestingly, in this model VR17-04 would form no or only one hydrogen bond with guanine or uridine (Figure 4A), suggesting that no stable Watson–Crick pair can be formed and that the inhibitory effect of VR17-04 would be dependent on the template sequence.

To investigate the hypothesis that VR17-04 could form base-pair interactions with the template in the SARS-CoV-2 nsp12/7/8 active site, we docked enisamium or VR17-04 into the SARS-CoV-2 nsp12/7/8 complex bound to template RNA and remdesivir monosphosphate (PDB 7bv2, Figure 4B). Prior to docking, we removed the remdesivir monosphosphate from the complex, and used in silico mutagenesis to change the now unpaired uridine in the template to cytosine or adenine. As shown in Figure 4C–E, we found that both enisamium and VR17-04 can be accommodated in the +1 position of the nucleotide binding pocket, in a position similar to remdesivir monosphosphate (Figure 4B). VR17-04 was specifically coordinated through hydrogen bond interactions with the unpaired cytosine or adenine base in the template RNA. In addition, our modelling suggests that VR17-04 can form a stacking interaction with the -1 base of the nascent strand. Nsp12 residues K545 and K555 were observed to play a role in coordinating VR17-04 in the catalytic cavity. By contrast, enisamium docked in the same +1 nascent strand position, but only formed one hydrogen bond with the cytosine in the +1 template position.

**Figure 5:**
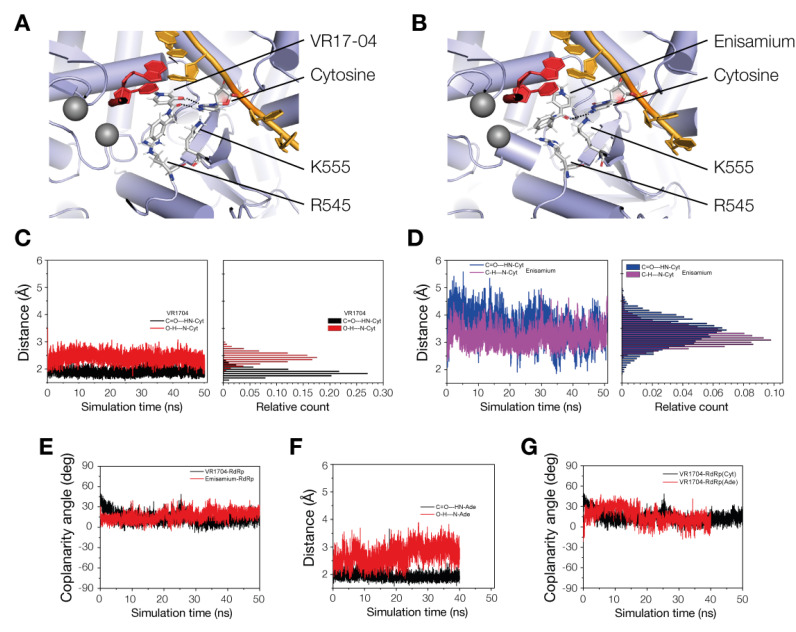
MD simulations of VR17-04 binding to nsp12/7/8. (**A**) MD simulation snapshot of VR17-04 binding to cytosine in nsp12 active site. Black dotted lines indicate hydrogen bonds. Template is shown in bright orange and nascent strand in dark red. Grey spheres represent magnesium ions. Positive charges on amino acids and base are dark blue and negative charges light red. Nsp12 is colored light blue and shown in a cartoon presentation. (**B**) MD simulation snapshot of enisamium binding to cytosine in nsp12 active site. Colors as in Figure 5A. (**C**) MD simulation plot of hydrogen bond distances during VR17-04 binding to cytosine in nsp12 active site (left) and histogram of hydrogen bond distances (right). (**D**) MD simulation of hydrogen bond distance (C=O---HN-Cyt, blue color) during enisamium binding to cytosine in nsp12 active site. Since no second hydrogen bond can form in the case of enisamium binding to nsp12/7/8, the distance C-H---HN-Cyt is reported in magenta color for comparison. (**E**) MD simulation plot of the coplanarity angle of VR17-04 or enisamium binding to cytosine in nsp12 active site. (**F**) MD simulation of hydrogen bond distances during VR17-04 binding to adenine in nsp12 active site. (**G**) MD simulation of coplanarity angle of VR17-04 binding to cytosine or adenine in nsp12 active site.

To estimate the binding stability of enisamium or VR17-04 in the nsp12 active cavity, we performed MD simulation of VR17-04 or enisamium docked in the nsp12/7/8 complex (Figure 5A,B; see Materials and Methods for specifics). Our MD simulations predict that VR17-04 binds more favorably to the unpaired cytosine in the +1 position of nsp12/7/8 complex than enisamium, maintaining two hydrogen bonds (Watson–Crick base pair) at standard distances (Figure 5C,D) and a low coplanarity angle χ (Figure 5E). This is particularly evident from the ~2-fold difference in distance between enisamium and cytosine compared to VR17-04 and cytosine (Table 3, Figure 5C,D), and a higher estimated Poisson–Boltzmann free energy for the enisamium binding (43.6 kcal/mol) compared to VR17-04 binding to either cytosine (-19.8 kcal/mol) or adenine (-14.8 kcal/mol) (Table 4). We observed similar hydrogen bond distances and dihedral angles in our MD simulations of VR17-04 binding of cytosine and adenine (compare Figure 5C,F, and see Figure 5G), suggesting that both may be able to bind in the nsp12/7/8 complex active site.

## 4. Discussion

The rapid global spread of SARS-CoV-2 necessitates the development of effective therapeutic interventions, and the most promising short-term strategy is to repurpose existing drugs. In this study we showed that enisamium, which is approved for use against influenza in 11 countries, can inhibit SARS-CoV-2 infection and RNA synthesis in vitro (Figure 1 and Figure 2). MD simulation analysis and in vitro activity assays suggest that VR17-04 reversibly binds the exposed cytosine or adenine, preventing GTP and UTP incorporation into the nascent RNA chain (Figure 4 and Figure 5). The inhibition of nsp12/7/8 by VR17-04 is in line with previous in vitro activity assays with purified nsp12/7/8 [15].

It was previously reported that enisamium inhibits the influenza A virus RNA polymerase activity in vitro with a relatively high IC_50_ value of 46.3 mM [13]. This inhibition was improved 55-fold by the addition of a hydroxyl group in the compound VR17-04 [13]. A similar result was found for the SARS-CoV-2 nsp12/7/8 complex in a comparable in vitro assay [15]. Remdesivir triphosphate is the active metabolite of remdesivir, which has shown promise in both cell culture and clinical trials as a treatment for SARS-CoV-2 infection [16]. We observed that the inhibitory effect of enisamium was more pronounced in NHBE cells than in Caco-2 cells, which is in line with previous influenza A virus experiments [13,17] and suggests that enisamium is more readily metabolized into VR17-04 in primary bronchial epithelial cells compared to adenocarcinoma cells.

Our docking and MD simulations suggest that VR17-04 can bind a template cytosine or adenine base in the active site of the SARS-CoV-2 RNA polymerase, forming a Watson–Crick base pair interaction. This hypothesis is supported by our NOE experiments performed in water, which indicate that VR17-04 can adopt an *eclipsed* conformation in solution (unbound state). We predict that the *eclipsed* conformation is compatible with a sequence-specific inhibition of the nsp12/7/8 RNA polymerase. Future activity and structural studies can be performed to further uncover the molecular mechanism by which VR17-04 and enisamium inhibit the SARS-CoV-2 RNA polymerase, or other RNA virus RNA polymerases.

Overall, our results strongly suggest that enisamium metabolite VR17-04 inhibits RNA synthesis by the SARS-CoV-2 nsp12/7/8 complex. Moreover, unlike remdesivir, enisamium does not require intravenous administration, which would be advantageous for its use outside a hospital setting. Together with observations that enisamium can inhibit other RNA virus infections, and DNA virus infections [17,18], these results here suggest that it can act as a broad-spectrum polymerase inhibitor in vitro.

## Figures and Tables

**Table 1:** Quantum chemical (DFT B3LYP/6-31G*) geometry optimization of VR17-04 and enisamium. The dihedral angles (φ_0,_ φ_1_, φ_2_, φ_3_) that define the conformation of VR17-04 and enisamium are reported. VR17-04 allows two conformations at φ_3_ *trans* and *eclipsed*, respectively. The estimated energies (E^B3LYP^) and zero-point energies (E^ZPE^) are reported in Hartree. The ground state energy (E^B3LYP^ + E^ZPE^) are reported in Hartree. The energy difference between the two conformations (*trans*, *eclipsed*) in VR17-04 correspond to 0.0129 Hartree or 8.095 kcal mol^−1^).

Compound	φ_0_	φ_1_	φ_2_	φ_3_	Conf.	E^B3LYP^ (Hartree)	E^ZPE^ (Hartree)	E^B3LYP^+ E^ZPE^ (Hartree)
VR17-04	80	44	148	*141*	*trans*	−802.2589	0.2720	−801.9869
VR17-04	83	9	177	*−3*	*eclipsed*	−802.2713	0.2715	−801.9998
Enisamium	77	27	170	−21	−	−727.0392	0.2672	−726.7720

**Table 2:** ^1^H chemical shift of the VR17-04 in water at T = 277 K acquired at 500 MHz. The labelled resonances are reported on the NOE spectra in Figure 4B.

^1^H Resonance.	HN	H2′	H6′	H5′	Ph	CH_2_	CH_3_
δ ppm	10.45	8.29	8.08	8.13	7.42/7.36	4.66	4.28

**Table 3:** Structural characterization of the interaction (Watson–Crick base pair) between VR17-04-cytosine (Cyt), enisamium-(Cyt), and VR17-04-adenine (Ade), described by average distances 〈d〉 between hydrogen bond donor and acceptor groups, and average values of the coplanarity angle 〈χ〉. The errors on the mean values are smaller than the last decimal digit. The selected distances and dihedrals are defined by the atoms in bold. The average interval is reported.

Complex	D	〈d〉 (Å)	χ	〈χ〉 (°)	Avg Interval (ns)
VR17-04-RdRp(C)	CO---H_2_N-Cyt, OH---:N-Cyt	1.9, 2.4	CO-HN-N:-HO	13	30–50
Enisamium-RdRp(C)	CO---H_2_N-Cyt	3.3	CO-HN-N:-HC	20	30–50
VR17-04-RdRp(A)	CO---H_2_N-Ade, OH---:N-Ade	1.9, 2.9	CO-HN-N:-HO	11	20–40

**Table 4:** Poisson Boltzmann free energy of binding 〈Δ*G_PB_^Bind^*〉 estimated as average value using the MMPBSA methods; the corresponding error on the mean is reported in brackets. The time interval for the average estimation is reported.

Inhibitor	Complex	Average MD Interval (ns)	〈Δ*G_PB_^Bind^*〉 (kcal mol^−1^)
VR17-04	VR17-04-RdRp(Cyt)	[46, 50]	−19.8(4)
Enisamium	Enisamium-RdRp(Cyt)	[46, 50]	43.6(5)
VR17-04	VR17-04-RdRp(Ade)	[28, 32]	−14.8(5)

## Data Availability

Protein expression constructs are available upon request.
